# C-terminal domain of SMYD3 serves as a unique HSP90-regulated motif in oncogenesis

**DOI:** 10.18632/oncotarget.2970

**Published:** 2015-02-12

**Authors:** Mark A. Brown, Kenneth Foreman, June Harriss, Chhaya Das, Li Zhu, Melissa Edwards, Salam Shaaban, Haley Tucker

**Affiliations:** ^1^ Department of Clinical Sciences, Colorado State University, Fort Collins, CO 80523, USA; ^2^ Coferon Inc., Stony Brook, NY 11791, USA; ^3^ University of Texas at Austin, Institute of Cellular and Molecular Biology, Austin, TX 78712, USA; ^4^ Abbvie, Worcester, MA 01605, USA

**Keywords:** HSP90, SMYD3, tumorigenesis, lysine methylation, histone modifications

## Abstract

The SMYD3 histone methyl transferase (HMTase) and the nuclear chaperone, HSP90, have been independently implicated as proto-oncogenes in several human malignancies. We show that a degenerate tetratricopeptide repeat (TPR)-like domain encoded in the SMYD3 C-terminal domain (CTD) mediates physical interaction with HSP90. We further demonstrate that the CTD of SMYD3 is essential for its basal HMTase activity and that the TPR-like structure is required for HSP90-enhanced enzyme activity. Loss of SMYD3-HSP90 interaction leads to SMYD3 mislocalization within the nucleus, thereby losing its chromatin association. This results in reduction of SMYD3-mediated cell proliferation and, potentially, impairment of SMYD3′s oncogenic activity. These results suggest a novel approach for blocking HSP90-driven malignancy in SMYD3-overexpressing cells with a reduced toxicity profile over current HSP90 inhibitors.

## INTRODUCTION

The histone code of post-translational modifications determines the level of chromatin accessibility to both transcription factors and polymerase complexes [1–3]. In this way, the SMYD family of histone methyltransferases (HMTases) plays critical roles in the modulation of transcriptional activity to impart normal cellular differentiation as well as oncogenic transformation. SMYD3 catalyzes trimethylation (me3) of H4-K20 [4], H4-K5 [5] and H3-K4 [6] and monomethylation of vascular endothelial growth factor receptor 1 [7]. These various histone methylation marks lead to altered expression levels of genes physically associated with the methylated histone. Indeed, SMYD3 has been strongly implicated as a proto-oncogene in hepatocellular, colorectal and breast carcinomas [8–12] by virtue of its high over-expression and promoter-associated polymorphisms specific to malignant cells.

HSP90 is a key chaperone involved in the proper folding of many cellular proteins and its deregulation is strongly implicated in a broad array of malignancies [13, 14]. At the same time, HSP90 has been implicated as a driver of evolution, either as a stabilizer of particular polymorphisms in coding and regulatory sequences of key proteins [15, 16] or as an inducer of heritably altered chromatin states [17], suggesting it has a significant role in epigenetic modification. This later role is more surprising, as HSP90's primary role is traditionally seen as a folding chaperone to a vast number of client proteins, including a myriad of epigenetic regulators. It remains an open question as to whether HSP90 has specific interactions with a select few epigenetic proteins through which the heritably altered chromatin states are cooperatively induced.

HSP90 has been the target of many novel cancer therapeutics. The most advanced of these function by occupying the ATP binding site, thus blocking the release of HSP90 substrates. Unfortunately, adverse side effects are the unintended consequence of eliminating the molecular chaperone activity of this broadly expressed and essential protein [18]. Altering the association of HSP90 with specific partners is seen as a potential, but challenging and as yet unsolved, approach toward mitigating these side effects. One line of thought is that altering the epigenetic functions of HSP90 without significantly altering its molecular chaperone function might lead to a better tolerated therapeutic outcome.

SMYD3 and HSP90 can physically interact, with HSP90 stimulating the basal HMTase activity of SMYD3 [9]. The relevance of this association in a cellular milieu and its association with the epigenetic roles of either of these proteins is, however, poorly characterized. The potential to connect both the physical associations and the epigenetic functions of SMYD3 and HSP90 has increased significantly with the almost concurrent publication of three independent crystal complexes of SMYD3 [19–21]. The SMYD3 structures revealed an overall compact architecture in which the N- and C-terminal portions of the “split-SET” domain (N-SET and C-SET) adopt a canonical SET domain fold and closely assemble with the MYND (Myeloid translocation protein 8, Nervy, and DEAF-1) zinc-binding and protein-protein interaction domain [22–24]. The structures also feature a previously uncharacterized, ~150 residue C-terminal domain (CTD) which is conserved in all SMYD paralogs except SMYD5. The CTD forms a superhelical 9 α-helical bundle which constricts the floor of the substrate binding site to a variable degree among the SMYDs [25, 26]. Based on structural overlays, the superhelical bundle appears to be a second protein-protein interaction domain, termed the tetratricopeptide repeat (TPR). TPRs facilitate a wide range of diverse functions and are composed of ~34 amino acids of roughly conserved sequence that invariably assemble into characteristic helix-turn-helix structures [27]. A previously documented interaction of HSP90 with the TPR of the cyclophilin FKBP52 [28, 29] implicates the CTD as the HSP90 binding motif for most human SMYDs [30, 31]. This model was suggested for SMYDs 2, 3, and 5 [32], but the cellular consequences of potential HSP90-SMYD interactions have not been addressed.

Herein, we investigate the structural and functional relationship between HSP90 and SMYD3 both *in vitro* and *in vivo*. We show that the CTD is essential for basal SMYD3 methyltransferase activity and establish a unique interfacial interaction for maximal HMTase induction by HSP90. We suggest that disruption of the association between SMYD3 and HSP90 may impact cellular differentiation and oncogenic transformation, providing a potential avenue for blocking HSP90-enabled malignancy with a reduced toxicity profile in SMYD3-overexpressing cells.

## RESULTS AND DISCUSSION

### The CTD is required for basal HMTase activity of SMYD3

**Figure 1:**
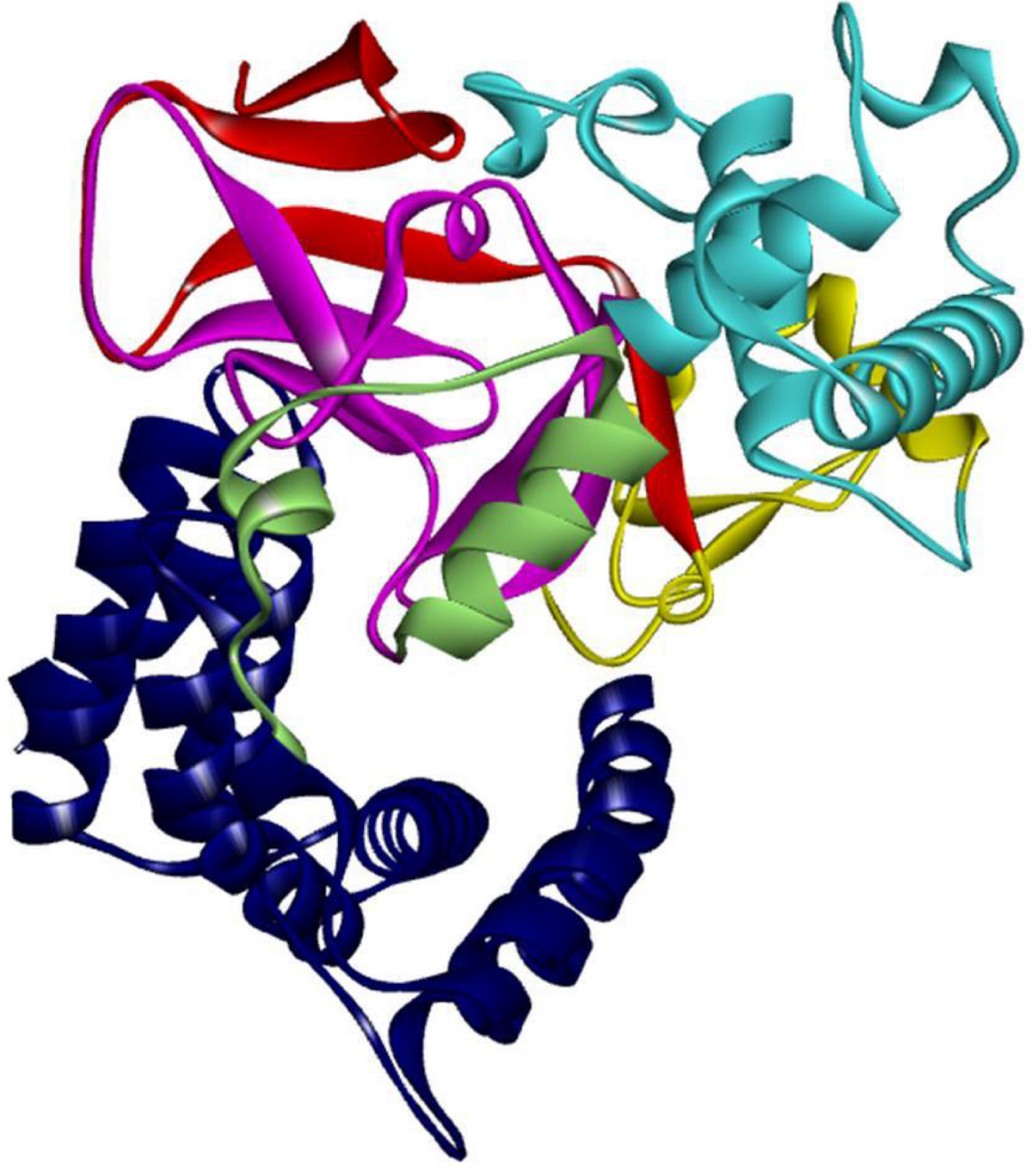
Structure of SMYD3 Structure of SMYD3 colored by domain components. SMYD3 has 6 domain components: N-SET (red), MYND (Yellow), I-SET (cyan), C-SET (magenta), post-SET (pale green), and the CTD (blue).

**Figure 2:**
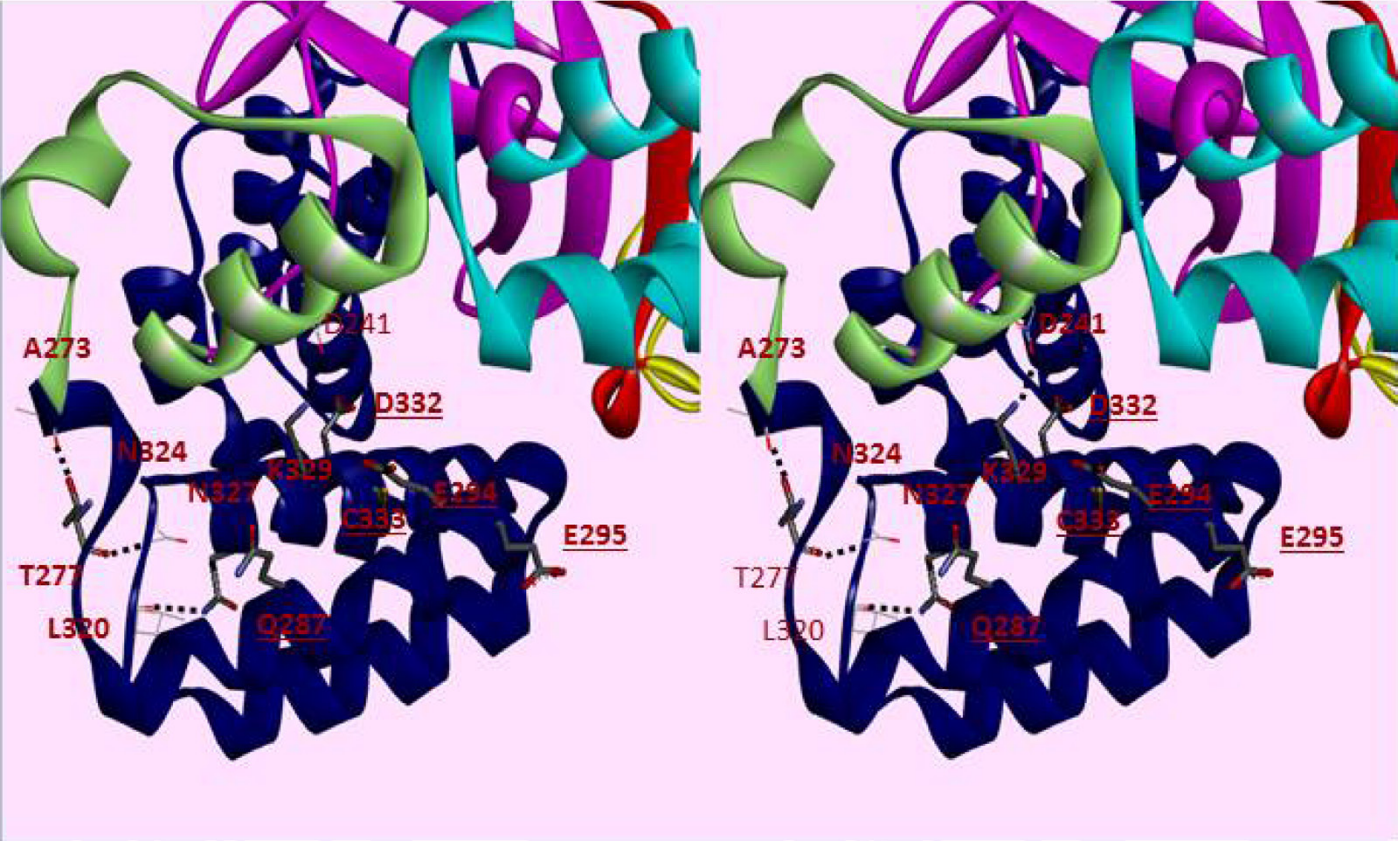
Cross-eye Stereo view of helices 1–6 of the CTD of SMYD3 SMYD3 domains are colored separately, with the CTD colored blue. Residues conserved in SMYD3 orthologs but not paralogs are displayed in thick bonds. Dashed lines indicate hydrogen bonds. Underlined residues are available on the surface for interactions. Other domains include the N-SET (red), MYND (yellow), I-SET (cyan), C-SET (magenta) and the post-SET domain (pale green).

**Figure 3:**
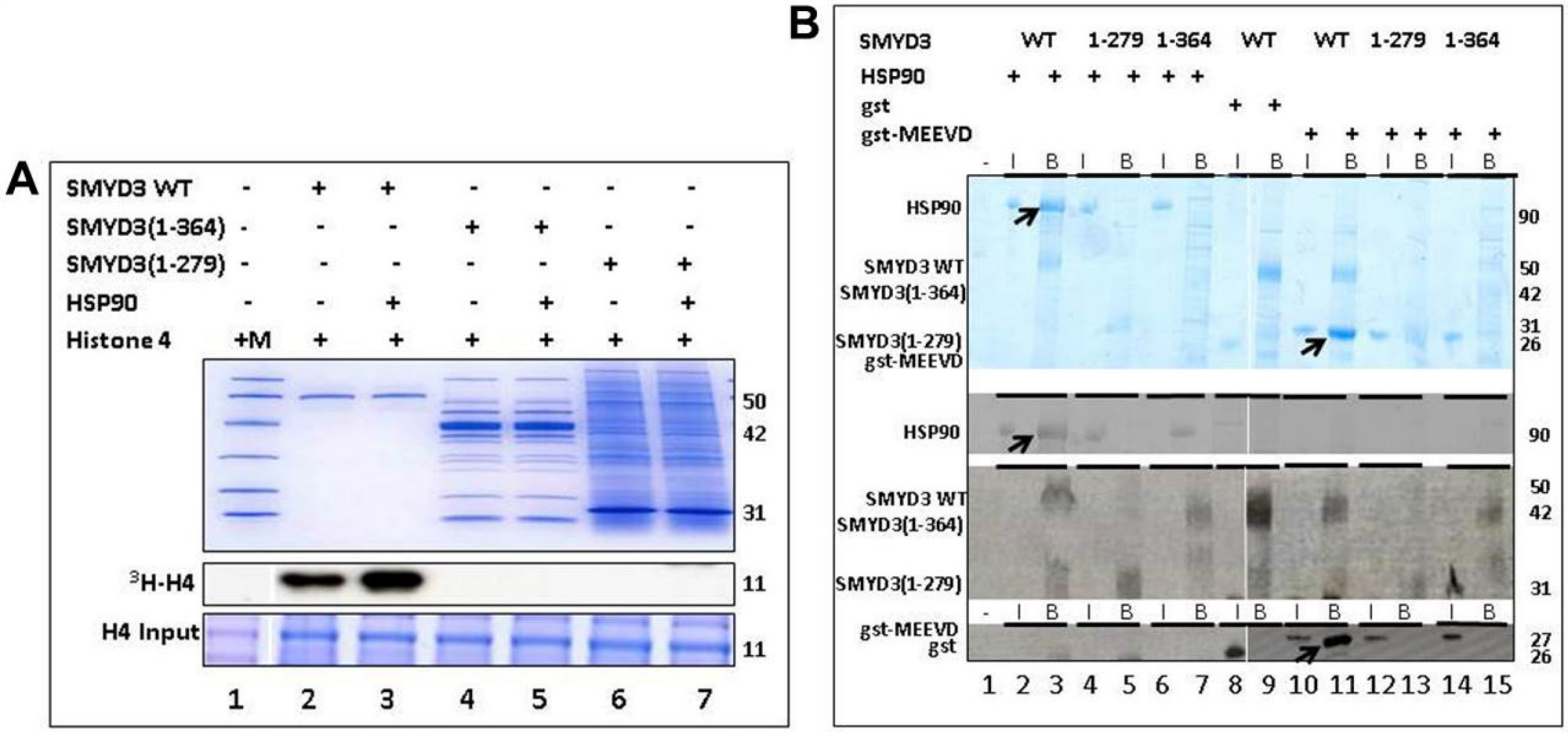
The SMYD3 CTD is required for binding and enhanced Histone Methyl Transferase Activity (HMTase) by HSP90 **(A)** Truncation mutants were expressed in *E. coli* and validated for purity on 10% SDS-PAGE stained with Coomassie Blue (Upper panel). Equal amounts of truncated and wildtype (WT) SMYD3 were then compared for *in vitro* HMTase activities in the presence or absence of HSP90 by ^3^H-S-adenylmethionine incorporation into histone H4 (^3^H-H4) followed by gel fractionation and autoradiography after loading onto a separate 20% SDS-PAGE (lower and middle panels). The SMYD3(1–279) truncation eliminates the entire CTD, while SMYD3(1–364) lacks the final 3 helices of the CTD. Molecular weights in kD indicated to the right of each panel were determined from marker mix (M, included in Lane 1 with H4 only) which, as indicated by the blue vertical line was run on parallel 10% and 20% gels. **(B)** The SMYD3 CTD is required for binding of HSP90 and for binding to a pentapeptide MEEVD previously shown [29] to be sufficient for the interaction of HSP90 and a TPR domain within the immunophilin, FKBP52. Nickel-NTA beads were mixed with ~1 μg wildtype (WT) 6X-His-SMYD3 or ~1 μg 6X-His-mutants in which the entire CTD (1–279) or its C-terminal 3 helices (1–364) were truncated. The slurries were incubated with either HSP90α or GST-MEEVD and bound protein (B lanes) was eluted from the beads and analyzed on 12.5% SDS-PAGE. For input controls (I lanes), 10% of the amounts of HSP90α and GST-MEEVD used for binding reactions were processed identically but in the absence of 6X-His-SMYD3. Band assignments (left) were made by sizes of Coomassie stained bands (upper panel) as judged by migration of a standard molecular weight marker mix (not shown). These assignments were confirmed (lower panels) by western blotting using antibodies (indicated on the left) specific for SMYD3, HSP90 and GST. Arrows denote positions of WT bound HSP90 or GST-MEEVD. Molecular weights are indicated on the right in kD. Blue vertical lines denote composites of lanes run on parallel gels repositioned to emphasize outcomes.

Inspection of the SMYD3 structure (Figure 1) revealed that a relatively large space near the post-SET domain and N-terminal portion of the CTD along the inner wall of the pocket is decorated by polar residues from the CTD (mainly residues from N324-C333 of helix 4) (Figure 2). Sirinupong et al. [20] had identified residue K329 as a key linchpin residue, helping maintain the spacing between the CTD and the rest of the protein. In addition, residues T277 and N327 form multiple hydrogen bonds which help stabilize the assembly of helices 1–4 of the CTD. The remaining residues (E294, E295, D332, and C333) all align in roughly linear fashion in close succession, except for Q287. This conserved clustering suggests that these polar residues might cooperate with the post-SET residues to restrict the histone substrate on both sides of the methyl-lysine. In this context, the CTD could function as a cap necessary to bind substrates effectively and selectively. Consistent with this hypothesis, deletion of CTD helices 1–9 [SMYD3(1–279)] eliminated basal HMTase activity of SMYD3 for histone H4 (Figure 3A).

This loss in basal HMTase is also associated with significantly reduced binding of SMYD3 to HSP90 (Figure 3B). The C-terminal five residues (MEEVD) of HSP90 are putatively sufficient to recognize TPR motifs [29]. While this pentapeptide bound WT SMYD3, it failed to interact significantly with SMYD3(1–279). This indicated that not only is the CTD required for the basal HMTase activity of SMYD3, but that recognition of HSP90 via its last five C-terminal residues may also be required. Unexpectedly, deletion of helices 7–9 [SMYD3(1–364)], which neither contains nor interacts with any of the polar residues mentioned above, also led to loss of basal HMTase activity and to loss of binding to HSP90 and its derivative MEEVD peptide (Figure 3).

### Structural conservation of SMYD3 CTD and the HSP90-binding tetratricopeptide (TPR) repeats within FKBP52

**Figure 4:**
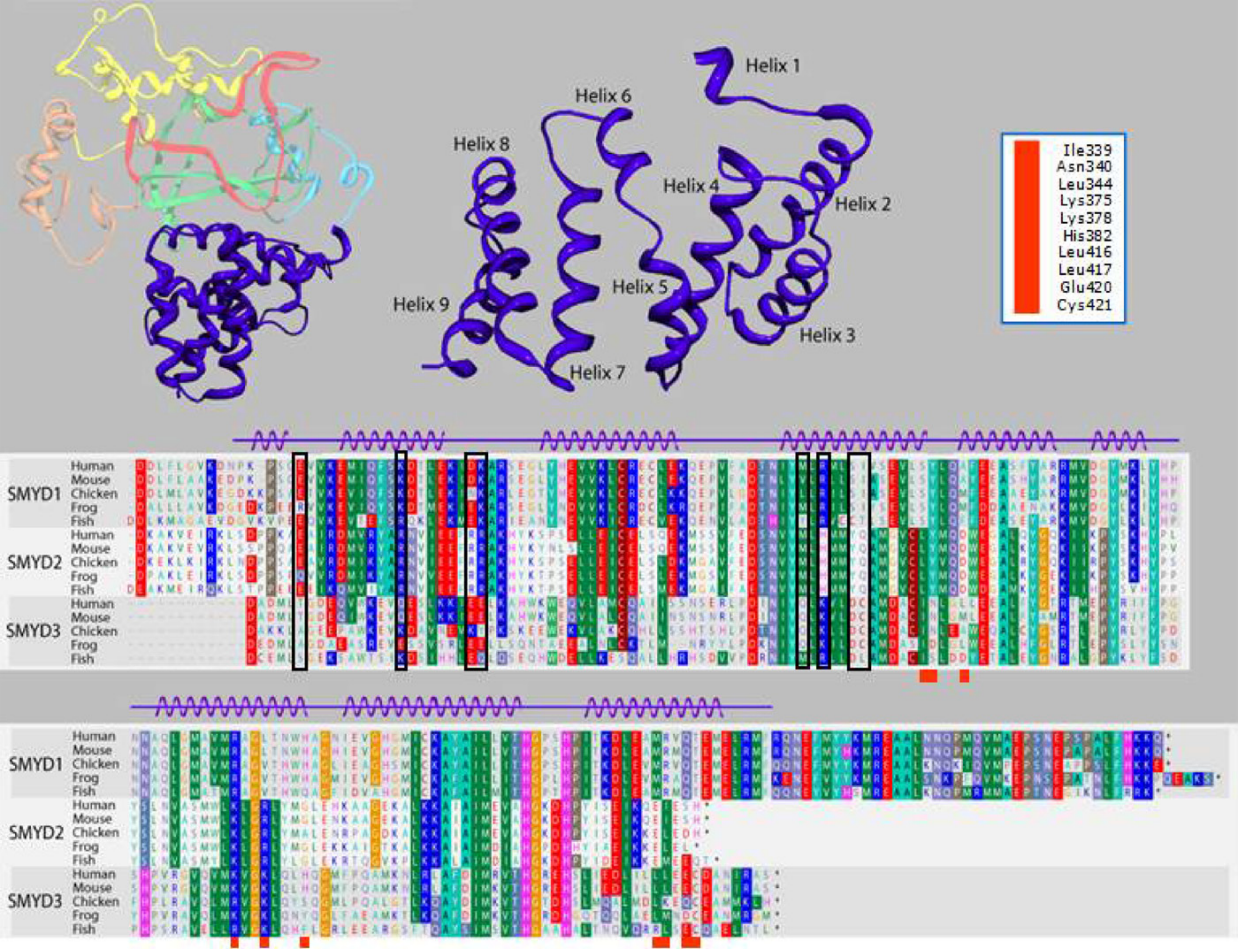
Alignment of the carboxyl terminal (CTD) domain from SMYD3 orthologs and paralogs Structure of the 9-helix bundle SMYD3 CTD (right upper panel, blue). The primary human sequence of SMYD3 CTD is aligned (lower panel) with corresponding CTDs of closest paralogs, SMYDs1 and 2, in multiple species. Residues are colored according to their physical properties. For example, all shades of red represent acidic residues, all shades of blue represent basic residues, and all shades of green represent hydrophobic residues. Black boxes in the alignment indicate residues conserved among orthologous SMYD subfamilies but not among paralogs in the same species. Red boxes below the alignments correspond to the SMYD3 residue labels in the upper right hand corner. These residues are modeled as being within 6Å of the MEEVD C-terminal peptide from HSP90 in human SMYD3 (see Figure 5B) or were mutated (see Table I).

**Figure 5:**
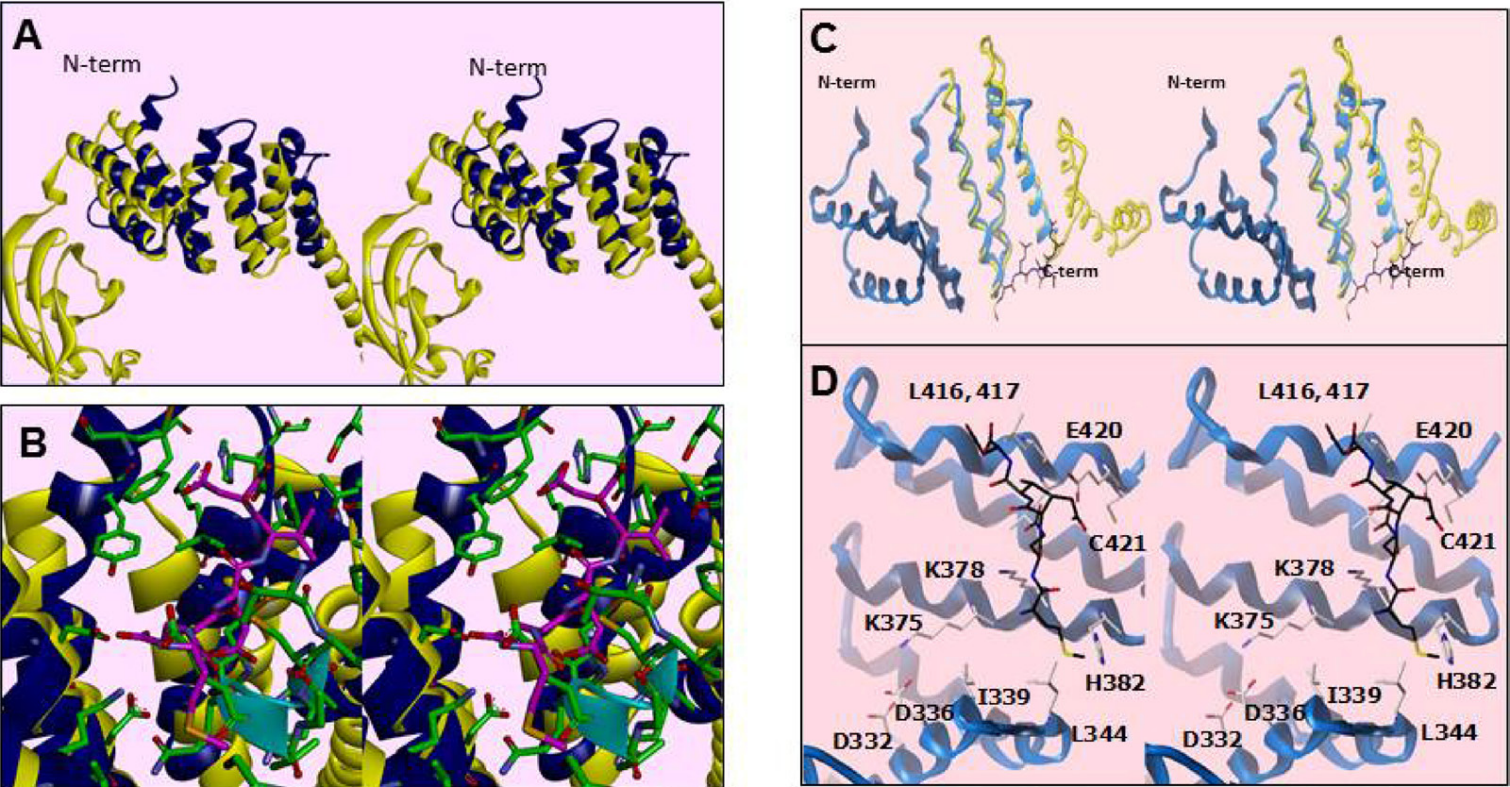
Residues within a degenerate tetratricopeptide (TPR)-like domain within the SMYD3 CTD mediate HSP90 interaction **(A)** Cross-eye stereo recapitulation of the modeled overlay of SMYD3 CTD (blue) with the TPR motif from FKBP52 (yellow) as in [20, 21, 26]. The N-terminal of the CTD is labeled for clarity of orientation. **(B)** Stereo close-up of the overlay in Figure 5A, with ribbon coloring retained. Residues from SMYD3 are in green while the MEEVD pentapeptide from the C-terminus of HSP90 in the FKBP52-bound structure is in magenta. The residues from the I-SET domain are marked by the cyan ribbon. **(C)** Stereo depiction of the current publication's modeled overlay of helices 7–9 of the SMYD3 CTD (blue) with the HSP90-binding region of FKBP52 (yellow). **(D)** Stereo close-up of the overlay in Figure 5C, with ribbon coloring retained. Residues from HSP90 are in black, while those from SMYD3 are in off-white. Residues in the vicinity of the modeled HSP90 peptide are labelled.

To reconcile the above results for SMYD3(1–364), a model of the binding of HSP90 to the CTD of SMYD3 proved extremely helpful. The CTDs are significantly conserved among SMYDs 1–3 and their orthologs after position 364 of SMYD3 (Figure 4). Others [20, 21, 26] have posited that the CTD of various SMYDs may be associated with HSP90 binding and have even generated overlays predicting the orientation of the MEEVD peptide in the TPR-like motif. Recapitulation of this overlay (Figure 5A, 5B) using FKBP52, which was solved in a complex with the terminal 5 amino acids (MEEVD) of HSP90 [29], indicated that the overlay may be incorrect. First, the HSP90 pentapeptide is inserted deep into the pocket, leading to a potential steric conflict between HSP90 and substrates of SMYD3. The HSP90 CTD is almost certainly not a disordered domain nor is it a purely linear chain. But in this model, the CTD must be positioned somewhere near the lip of the SMYD3 protein, thereby reducing access. Second, the residues in that region are incompatible with the HSP90 peptide (Figure 5B). Several of the residues of the MEEVD peptide model are in steric clash with the CTD residues, where a loop from the I-SET domain occupies a similar space. This clash was rationalized away by hypothesizing an autoinhibitory mode [26], which we grant is possible. Yet, even if the SMYD3 side chains were adjusted so as to relieve steric clashes, the acidic residues of the HSP90 C-terminal tail sit in a neutral to acidic portion of the pocket, suggesting a lack of electrostatic complementarity as well. Third, deletion of helices 7–9 should not significantly perturb the MEEVD peptide binding which is inconsistent with our data (Figure 3B). Thus, an alternative binding mode must be considered.

### TPR-like residues of SMYD3 CTD are essential *in vitro* for HSP90 binding and catalytic enhancement

**Figure 6:**
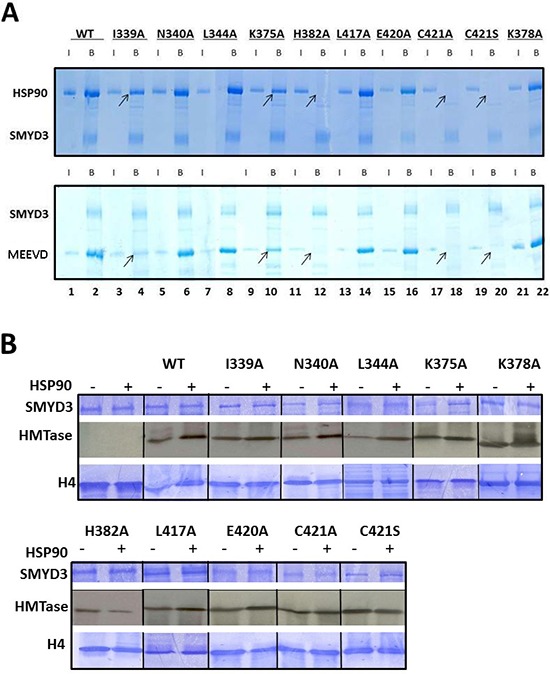
Enhancement of basal HMTase activity of SMYD3 requires binding of HSP90 to conserved residues within a TPR-like region of the CTD **(A)** Conserved residues within a TPR-like region of SMYD3 CTD are required for HSP90 and MEEVD binding. Residues mutated (detailed in Materials and Methods) were predicted to interact with the HSP90 peptide or to be critical for CTD integrity. Nickel-NTA beads were mixed with ~1 μg wildtype (WT) 6X-His-SMYD3 or ~1 μg 6X-His-mutants. The slurries were incubated with either HSP90α or GST-MEEVD and bound protein was eluted from the beads and analyzed on 12.5% SDS-PAGE. Arrows denote loss of SMYD3-mutant interaction. Blue vertical lines denote repositioning of lanes run on the same gel repositioned to emphasize outcomes. **(B)** CTD residues required for HSP90 binding are required for HSP90-mediated enhancement of SMYD3 HMTase activity. *In vitro* ^3^H-SAM HMTase assays (autoradiographs, center panels) were performed as described in Materials and Methods and in the legend to Figure 3A. Inputs are shown by Coomassie stains in upper (SMYD3 WT and mutants) and lower (recombinant histone H4) panels. Blue vertical lines denote repositioning of lanes run on the same gel repositioned to emphasize outcomes.

In order to reconcile our data with a TPR-like motif which could bind HSP90, we aligned helices 7–9 with the HSP90 binding region of FKBP52 [28, 29] (Figure 5C). Helices 4–9 in SMYD3 align with the first 3 helices of the TPR motif from FKBP52 (Figure 5D), which features the C-terminal pentapeptide MEEVD. Several SMYD3 residues between CTD helices 4 and 5, 7 and 8, and at the end of helix 9 were predicted from the FKBP52 structure [29] to be within contact distance (6Å) of the modeled HSP90 pentapeptide. All are conserved among closest SMYD3 orthologs and, to varying degrees, among SMYD3 paralogs and with FKBP52 (Figure 4; Table 1). Mutation of several of these residues led to diminished SMYD3 binding to both HSP90 and MEEVD, including I339 and K375, structural anchors between N- and C-terminal components of the CTD, or to complete loss of binding on mutation of H382, a potential HSP90 interfacial residue, or C421, an anchor for helix 9 to the rest of the CTD (Figure 6A). Dissociation constant (K_d_) measurements averaged from 5 biologic replicas with density measurements within linear range (Materials and Methods) indicated that HSP90 and MEEVD binding losses ranged from ~10-fold for I339A at the low end to ~40-fold for C421A at the high end (Table 1). The same point mutations lost up to 8-fold enhancement of HSP90 stimulation of HMTase activity toward histone H4 (representative data in Figure 6B; Table 1).

**Table 1:** Summary of HSP90 binding and histone methyltransferase activities following truncation or point mutation of residues within the SMYD3 CTD

SMYD3	conserved in SMYDs/FKBP52^§^	induced/basal HMTase^#^	K_d_(μm) HSP90	K_d_(μm) MEEVD	*p* value
WT		4.8 ± .0.7	18 ± 4	55 ± 12	< .001
1-279		< 0.1	>1000	>1000	< .001
1-364		< 0.1	>1000	>1000	< .001
I339/A^†^	1, 2, 3, F	2.4 ± 0.5	352 ± 29	544 ± 88	< .05
N340/A***	1	3.0 ± 0.4	39 ± 14	78 ± 14	
L344/A**, ***	1	3.8 ± 0.7	14 ± 7	85 ± 19	
K375/A^†^	1,2,3	1.1 ± 0.2	526 ± 90	670 ± 122	< .01
K378/A**, ***	2,3,F	3.3 ± 0.6	31 ± 4	33 ± 13	
H382/A**, ***	1,3,F	0.6 ± 0.4	777 ± 63	868 ± 94	< .01
L417/A**, ^†^	2,3,F	3.2 ± 0.5	96 ± 19	40 ± 10	
E420/A**, ***	1,3,F	3.9 ± 0.6	48 ± 16	30 ± 8	
C421/A^†^	1	1.0 ± 0.2	710 ± 132	850 ± 77	< .01
C421/S^†^	1	0.6 ± 0.3	622 ± 111	932 ± 205	< .005

§ Mutated residues conserved among SMYDs 1, 2, 3 and/or FKBP52 (F)

# ratio of HSP90-induced- to basal- *in vitro* SMYD3 HMTase activities for the indicated construct (Methods and Materials). A minimal number of 4 biological replicates were measured to determine ratios, standard deviations, and statistical significance. Basal levels were eliminated by the two CTD deletions [SMYD3 (1-364) and (1-279)] but remained essentially unchanged for any of the point mutations listed here.

** SMYD3 side chain predicted to be within 6Å of bound HSP90 pentapeptide, MEEVD

*** SMYD3 surface residue

† Critical SMYD3 structural element.

To ensure that these mutations are specific, we mutated nearby residues, such as N340 and E420. These mutations had no effect on HSP90 binding or enhancement (Table 1). Taken with the data of Figure 4, these results indicate that the more N-terminal helices of the SMYD3 CTD are required for its constitutive HMTase activity, whereas the TPR-like C-terminal helices are required for the enhanced activity afforded by HSP90.

### TPR-like residues of SMYD3 CTD are essential *in vivo* for nuclear localization, HSP90 interaction and sub-nuclear sequestration into chromatin

**Figure 7:**
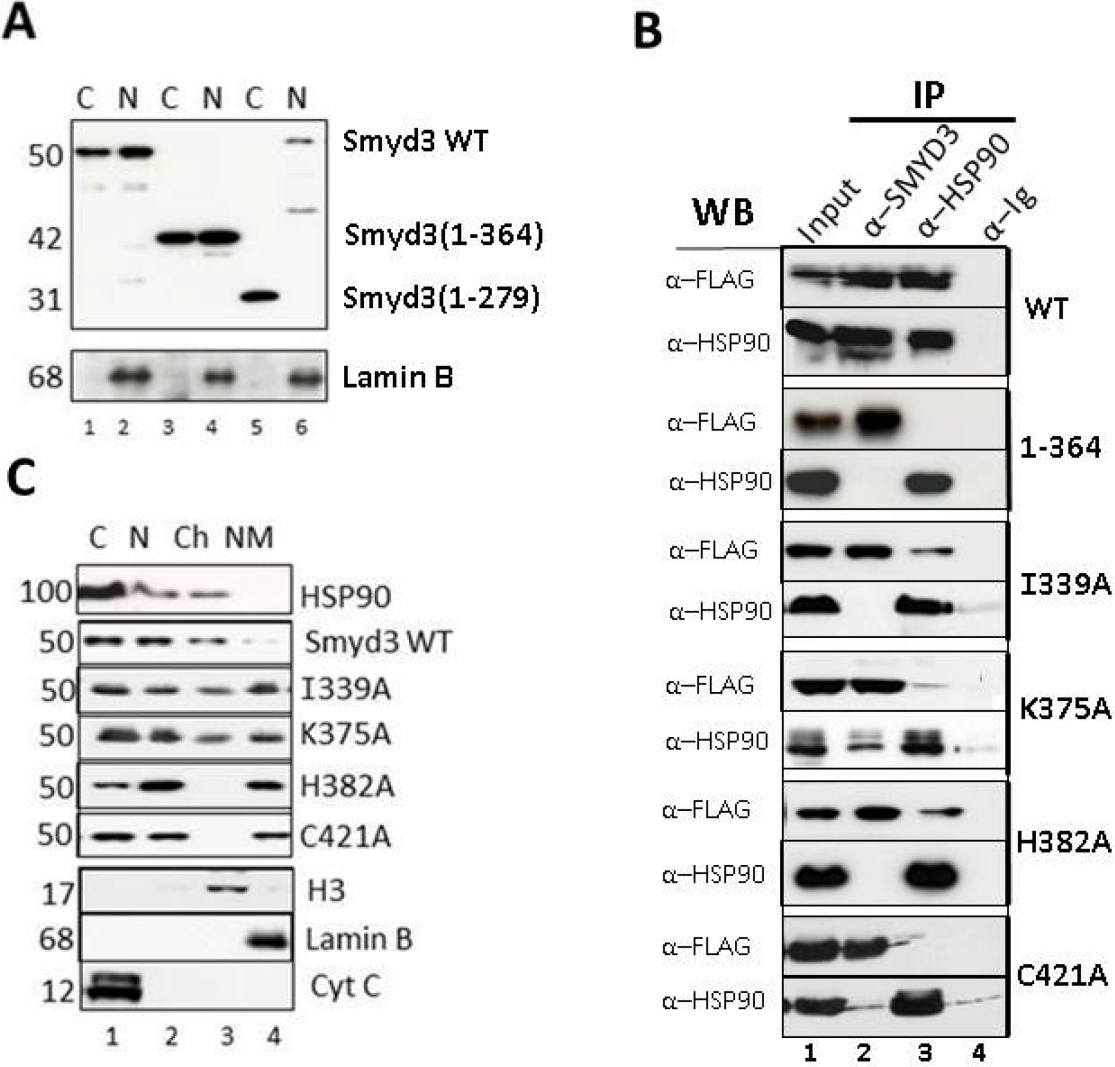
TPR residues of SMYD3 CTD are essential *in vivo* for nuclear localization, HSP90 interaction and sub-nuclear sequestration into chromatin **(A)** Deletion of the 9 helices of the CTD eliminates nuclear localization. Upper panel: NIH3T3 cells were transiently transfected with the indicated FLAG-tagged SMYD3 WT or deletion mutants, separated into nuclear (N) and cytoplasmic (C) fractions, and protein lysates were analyzed by SDS-PAGE/anti-FLAG western blotting. Purity of the fractions and confirmation of equal protein inputs was confirmed by anti-Lamin B western (lower panel). **(B)** Confirmation *in vivo* of SMYD3-HSP90 interactions established *in vitro*. Following transient transfection of the indicated FLAG-tagged wildtype (WT) and CTD point mutants into NIH3T3 cells, ~5% of the whole cell lysate was reserved for Input (lanes 1) and the remainder was subjected to immunoprecipitation with antibodies (α) specific for SMYD3 or HSP90 (IP; lanes 2 and 3) with pre-immune sera (anti-Ig, lanes 4) serving as negative control. Complexes were resolved on SDS-PAGE, and interactions of over-expressed SMYD3 with endogenous levels of HSP90 were assessed by anti-HSP90 and anti-FLAG western blotting. **(C)** CTD mutation perturbs distribution of SMYD3 within sub-nuclear compartments. Following over-expression of the indicated FLAG-tagged constructs, NIH3T3 cells were fractionated into cytoplasmic (C), soluble nuclear (N), chromatin (Ch) and nuclear matrix (NM) and following resolution on SDS-PAGE, protein subcellular localization was assessed by semi-quantitative anti-FLAG Western analysis. Western blotting with established markers (indicated in bottom 3 panels) validated purity of the sub-fractions.

To establish the cellular effects of the deletion and point mutants which impaired HSP90 binding *in vitro*, nuclear (N) and cytoplasmic (C) distributions of their overexpressed FLAG-tagged constructs were evaluated in NIH3T3 fibroblasts. As shown in Figure 7A, deletion of the 9 helices of the CTD in SMYD3(1–279) eliminated nuclear localization (compare lanes 5 and 6), whereas deletion of helices 7–9 in SMYD3(1–364) (lanes 3 and 4) showed no difference with wildtype (WT, lanes 1 and 2). Thus, nuclear entry function resides within helices 1–6 of the CTD. Potentially relevant is the previous observation that the predictive general nuclear localization sequence (NLS) for Kapβ2 transporter recognition (ΦGΦΦX_13_RX_3_PY; Φ, any hydrophobic residue) [33] matches the SMYD3 sequence from L341 to Y358. This sequence and, particularly the P357Y358 essential for Kapβ2-NLS recognition, are not exposed, but buried by helices 7–9 of the CTD of SMYD3, suggesting HSP90 C-terminal binding may serve to expose the putative NLS.

Next we tested whether the HSP90 binding requirements established *in vitro* were observed in cells. Following over-expression of the indicated SMYD3 constructs of Figure 7B, ~5% of the protein was reserved for Input (lane 1) and the remainder was subjected to anti-SMYD3 or anti-HSP90 immunoprecipitation (IP; lanes 2 and 3) with pre-immune sera (α-Ig) serving as a control (lane 4). Complexes were resolved on SDS-PAGE, and interactions of over-expressed SMYD3 with endogenous levels of HSP90 were assessed by anti-HSP90 and anti-FLAG western blotting. Strong, reciprocal interaction was observed for SMYD3 WT, whereas no interaction was detectable if helices 7–9 were truncated [SMYD3(1–364), Figure 7B]. Each of the SYMD3 point mutants which had reduced or no interaction with HSP90 *in vitro* (Figure 6B) showed highly reduced interactions in NIH3T3 cells (Figure 7B).

The lack of association between HSP90 and SMYD3 mutants raised the possibility that HSP90 interaction with SMYD3 CTD, and particularly helices 7–9, is essential for SMYD3 nuclear transport. To address this, we over-expressed select SMYD3 substitution mutants (Figure 7C), fractionated the NIH3T3 cells into cytoplasmic (C), soluble nuclear (N), chromatin (Ch) and nuclear matrix (NM) components and then carried out semi-quantitative anti-FLAG Western analysis. Established markers (bottom 3 panels) validated purity of the sub-fractions. As previously shown [34, 35], HSP90 accumulates in the cytoplasm (C) and within the soluble and chromatin sub-fractions (Figure 7C, lanes 1 and 3). WT SMYD3 accumulated in a similar pattern as HSP90. While nuclear localization was achieved with SMYD3(1–364) and each of the non-HSP90 interacting point mutants, they were mislocated to various extents, with virtually complete loss of K375A and H382A from chromatin into the nuclear matrix (compare lanes 3 and 4). Hence, association of the SMYD3 CTD with HSP90 is not required for nuclear transport per se but is required to distribute SMYD3 to its site of functional catalysis-nuclear chromatin.

### CTD-HSP90 interaction is required for maximal SMYD3 stimulation of cell proliferation

**Figure 8:**
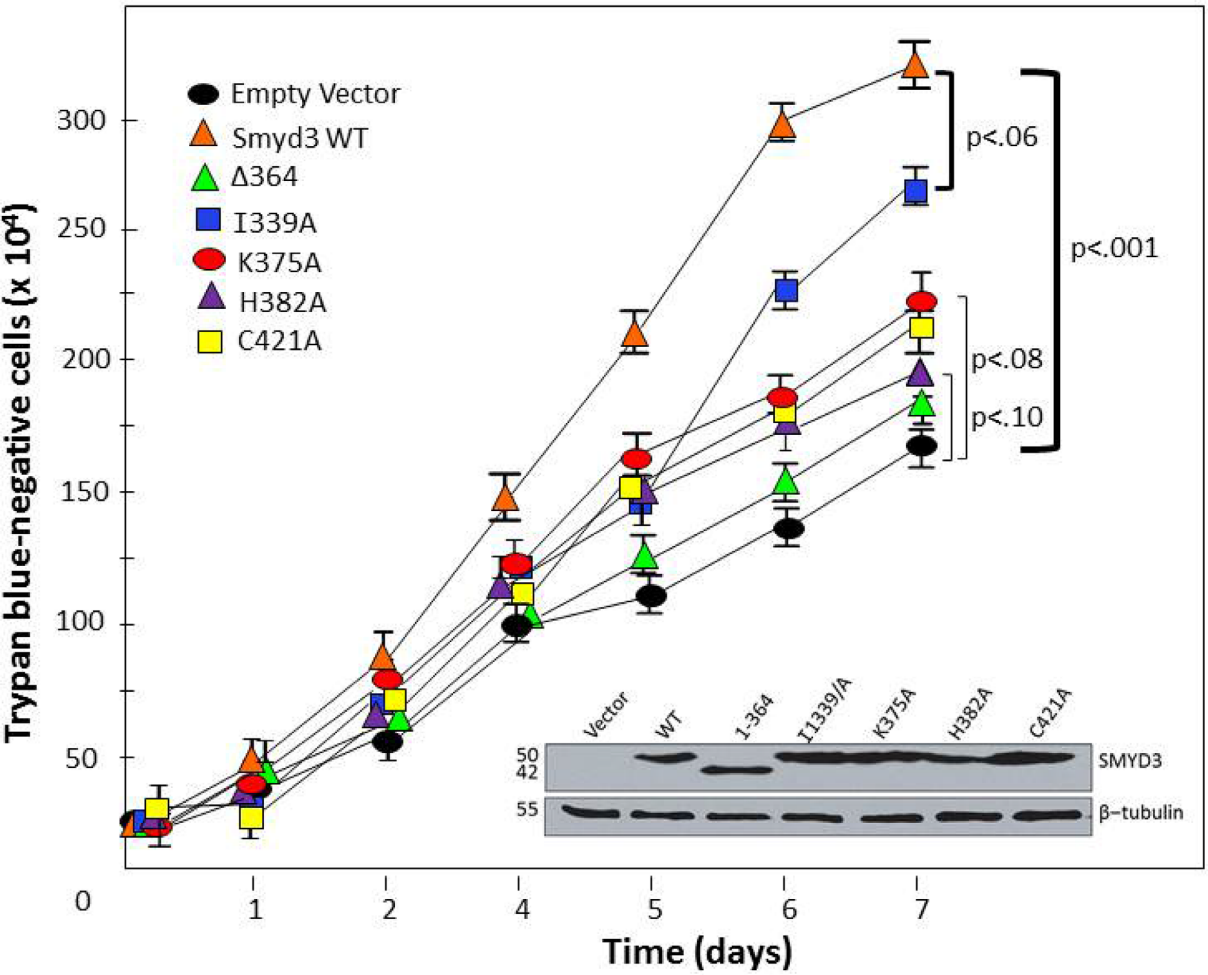
CTD-HSP90 interaction is required for maximal SMYD3 stimulation of cell proliferation rate The indicated WT and mutant constructs (top left) were transiently transfected into mouse embryonic fibroblasts. Relatively equal levels of expression were confirmed by Western blot of total cell lysates at day 1 (inset). Proliferation rates were assessed at the indicated time-points following transfection by counting trypan blue-negative (living) cells. Growth curves are shown as averages of 4 independent experiments with standard deviations (I) Brackets denote paired t-test-derived mean-difference probabilities (p) with width of the bracket representing magnitude. Δ364 stands for SMYD3 (1–364).

Although maximal nuclear activity of SMYD3 requires HSP90 association, its activity against cytoplasmic targets may be uncompromised and hence may not require HSP90 interaction for its oncogenicity. Numerous studies [6, 10, 36–38] have demonstrated proto-oncogene-type actions of SMYD3 under conditions of genetic-based promoter mutations leading to gain-of-function in malignant tumors or following enforced ectopic over-expression in non-transformed cells. HSP90 assists in the folding and function of numerous proto-oncogenes, as its inhibition by small molecules or siRNA leads to their destabilization and subsequent suppression of malignancy. As shown in Figure 8, stable over-expression of wildtype SMYD3 in mouse embryonic fibroblasts (MEFs) leads to a statistically significant (*p* < .001) approximately 3-fold enhancement in proliferation relative to vector control. This enhancement is significantly abrogated to varying extents in SMYD3 CTD mutants impaired in HSP90 interaction. Specifically, we observed low statistical difference (*p* < 0.10) between vector-only and all CTD mutants which lose HSP90 association, whereas the I339A mutation which retains HSP90 association trends much more closely to that of WT SMYD3 (*p* < 0.10). We did not observe significant changes in morphology, adhesion or cell migration following enforced expression of SMYD3, as was observed in some previous reports [6, 10, 36–40]. That these previous enforced expression studies were performed in transformed cell lines, which quite probably express higher levels of endogenous SMYD3 than did our diploid MEF transfectants, may account for this difference.

## CONCLUSIONS

SMYD3 is overexpressed in a variety of tumor types, including hepatocellular carcinomas and breast cancers, with poor prognosis commonly observed [41]. It is an important epigenetic regulator, known to methylate histones at several sites, including H4-K20. We demonstrate that the C-terminal domain (CTD) is essential for SMYD3's histone methyltransferase (HMTase) activity, as truncates of either the whole CTD or even just the three C-terminal helices of the CTD suffice to eliminate basal methylation of H4, both *in vitro* and *in vivo*. A central hypothesis proposed in the analysis of SMYD1 as applied to SMYD3 [20] conflicts with our results. Based on the differential geometries adopted by the CTDs of SMYD1 and SMYD3, those authors speculated that the SMYD3 CTD must undergo a hinge-like movement to relieve its inherent auto-inhibition of substrate entry and/or release, suggesting that the CTD serves mainly a regulatory role. In contrast, we directly demonstrated that CTD deletion greatly reduced enzymatic activity (Figure 3A and Table 1), suggesting that, at least for basal histone catalysis, the CTD stabilizes the SMYD3 active site.

To further understand the binding determinants of this regulatory site, we hypothesized that HSP90, which is known to stimulate SMYD family HMTase activity upon binding, interacts with the final three helices of the CTD. This proposal stands in contrast to earlier predictions which suggested that the entire CTD should interact with HSP90, based on overlays between the CTD and the HSP90-interacting tetratricopeptide repeat (TPR) domain of FKBP52. Detailed analysis of the HSP90 binding site modeled onto SMYD3 suggested several residues important for binding. These residues are conserved among both SMYD3 paralogs and orthologs, indicating potential functional significance. Our data establish a strong correlation between modification of these terminal helices, either through truncation or through point mutations, and sensitivity to HSP90 activation. Most strikingly, the side chain of H384 points out into solvent and is not expected to play a role in SMYD3 CTD conformational integrity but is predicted in our model to play an important role in HSP90 C-terminal recognition. As predicted, the H384A substitution mutant suffers near complete loss of HSP90 activation of SMYD3 both in *in vitro* and cellular contexts, suggesting the modeled binding mode is indeed predictive. Only the last 5 C-terminal residues of HSP90 (MEEVD) play a significant role in SMYD3 activation, as association and activation patterns are nearly identical between the full length HSP90 and the 5-mer (Table 1).

The TPR-like CTD of SMYD3 also appears necessary for cell localization and for nuclear trafficking. Indeed, the TPR motif has been well documented for its role in HSP90-dependent protein localization [42]. For example, mitochondrial localization of the immunophilin FKBP51 is dependent upon HSP90 via the TPR motif of FKBP51 [43]. Without a TPR motif and/or in the absence of HSP90, FKBP51 translocates to the nucleus where it has been shown to prevent oxidative stress [43]. Conversely, the association of many nuclear hormone receptors with TPR-motif containing proteins is known to facilitate their transport into the nucleus via association with HSP90 complexes [42]. Such is the case with the mineralocorticoid receptor which is transported into the nucleus by way of its association with TPR-containing FKBP52 in an HSP90 complex [44]. Further investigation of the unanticipated role of the SMYD family's TPR-like CTD in intra-nuclear trafficking may provide insight into the potential for more specific localization of proteins regulated by the TPR-HSP90 interface. Experiments involving HSP90 chaperone inhibitors, such as geldanamycin, together with cells expressing either WT or mutant SMYD3 proteins, might lead to a better understanding of the interplay between these proteins. Significant research, however, still remains in order to fully delineate the influence of HSP90 conformation and activation state on the ability of its C-terminus to interact with TPR and TPR-like motifs, as well as its ability to influence cell localization and nuclear trafficking. Additional experiments which isolate the nuclear localization sequence of SMYD3 and its transporter would also be of value.

Our results indicate that a substantial, if not exclusive, component of SMYD3-driven proliferation derives from its CTD interaction with HSP90. HSP90 facilitates SMYD3 localization with chromatin and generically prevents SMYD3 destabilization. While we cannot exclude HSP90 catalyzed stabilization of SMYD3 as the primary driver of the proliferation results, the fact that MEEVD suffices to activate SMYD3 *in vitro* but is not considered relevant to HSP90's chaperone function suggests HSP90 most likely serves to relieve the regulatory components on SMYD3 to enable SMYD3's epigenetic function. Nevertheless, sorting out the relative importance of these multiple HSP90 interplays with SMYD3 in native and oncogenic environments warrants further exploration.

Based, then, on our data, a model of SMYD3-HSP90 cooperatively in heritably altering chromatin states emerges, with HSP90 interactions with the CTD of SMYD3 proceeding via a two-component regulatory motif. The terminal helices 7–9 in this motif serve as a regulator of both the nuclear localization and compartmentalization sequences, with regulation of the latter facilitated through the enhanced accessibility of histone substrates. Relief of this HSP90-dependent regulatory feature permits a conformation in SMYD3 that supports efficient substrate binding. The remainder of the CTD (helices 1–6) serves as a binding enhancer and specificity determinant for SMYD3 substrates. Increasing levels of SMYD3 in the presence of HSP90 effectively allows HSP90 to transform into an epigenetic agent.

Given the general nature of HSP90 as a stress sensor, the connection between HSP90 and SMYD3 offers a unique opportunity for insight into oncogenesis and possibly evolution. Since most cancerous cells are in a perpetually stressed state, teetering on the brink of apoptosis, they face continual selection pressure, much like evolving organisms do. Activation of epigenetic stress response pathways should permit those cells to access survival mechanisms that might not otherwise be accessible under lower stress conditions. Generically, oncogenesis and metastasis require the manipulation of several processes, such as metabolic and cell cycle checkpoints, apoptosis, expression/repression of cell adhesion and motility factors, and recruitment of angiogenic factors. The scope of this process is analogous to measures that are required for re-setting a differentiated cell to a state of pluripotency, followed by selection of another differentiated state, and favors the conditions required for rapid mutagenesis and micro-evolution. HSP90, also termed the ‘cancer chaperone’, has a central role in these processes by maintaining the stability and activity of many client proteins which are essential for each process [13]. SMYD3 appears to place suppressive marks in normal cells, but may inappropriately place activating marks on H3-K4 over time when continually overexpressed. This role reversal may occur because, even though SMYD3 has greater affinity for the H3-K4 site than for other histone peptide sequences, the specificity of its MYND domain partners prevent it from interacting significantly with those sites in normal cells. Overexpression may lead to saturation of those partner binding sites which would then permit SMYD3 recognition of these alternate high affinity sites. HSP90 putatively helps stabilize, localize, and activate the excess SMYD3, allowing continuous rewiring. Such expression levels would be permanently achieved by the types of malignancy-associated SMYD3 promoter polymorphisms previously observed [11, 12, 45], leading to rapidly proliferative clonal expansion well beyond what we observed in our data of Figure 8. Clearly, the ability to prevent reversion to a more pluripotent state in the first place may suffice to significantly reduce the short term threat from cancers, suggesting the interaction between HSP90 and epigenetic proteins such as SMYD3 needs a closer inspection.

The CTD mediated stability of the SMYD3 active site also implies that the CTD is a potential pharmacologic target for the selective knockdown of SMYD3. Most HMTases share a sizable affinity for the methyl donor, S-adenosyl-methionine (SAM), making such a site less desirable as a drug target. Small molecule inhibitors which target the substrate binding site of other HMTases have achieved reasonable potencies and selectivities against those HMTases [46, 47]. That even the most distal portions of the CTD are necessary for basal function, despite predictions of less direct involvement in substrate binding, implies a non-competitive, allosteric means to regulate SMYD3 activity.

The cooperation between HSP90 and SMYDs in oncogenesis presents a novel direction for the clinical management of the resulting malignancies from an HSP90 perspective as well. If its association with SMYD3 is a primary driver of its oncogenecity rather than its stabilization of other overexpressed oncogenic proteins, blocking that association could have dramatic effects. HSP90 has been the target of many novel cancer therapeutics which eliminate its chaperone function. Unfortunately, unintended consequences of eliminating the chaperone activity of this broadly expressed protein include off-target toxicities, such as a variety of gastrointestinal side effects [48]. The development of a drug which blocks HSP90-SMYD3 interactions via binding the CTD of SMYD3 may remove transformative avenues of HSP90-driven malignancy without inducing the unintended side effects associated with broad spectrum HSP90 chaperone inhibition. Such an inhibitor does not yet exist, but it would also still allow basal signaling of SMYD3 in the cytoplasm, thus affecting its nuclear signaling selectively. To ascertain the utility of this approach, development of probe compounds which target SMYD3 and specifically compete with MEEVD or which lock away the NLS is needed.

## MATERIALS AND METHODS

### Mutagenesis, cloning, and bacterial expression

Point mutants were generated using GeneEditor *in vitro* Site-Directed Mutagenesis System (Promega) according to manufacturer's instructions using as template full length human SMYD3 cloned into Gateway pENTR vector (Invitrogen). For PCR, samples were heated to 94°C for 5 min, subjected to amplification for 16 cycles of 0.5 min at 94°C, 0.5 min at 55°C, and 0.5 min at 68°C and extended after the last cycle at 72°C for 7 min.

### Bacterial protein purification

Polyhistidine (6xHis)-tagged SMYD3 wildtype, truncation and substitution mutants were shuttled using directional TOPO cloning into Gateway (Invitrogen) pET™-DEST42. High level expression was induced by IPTG in *E. coli* strains MG232 (Scarab LTM) or Hsp90Plus™ (Expression Technologies Inc). Cells were lysed in buffer A [50 mM Tris-HCl pH7.7, 250 mM NaCl with protease inhibitors (Roche Applied Science, Cat. #11–873-580–001)] and centrifuged to remove cell debris. The soluble fraction was purified over an IMAC column charged with nickel (GE Healthcare, NJ), and eluted under native conditions with a step gradient of 10 mM, then 500 mM imidazole. Proteins were then further purified by gel filtration using a Superdex 200 column (GE Healthcare, NJ), into 25 mM Tris-HCl pH7.6, 150 mM NaCl, and 1 mM TCEP. Protein was pooled based on SDS-PAGE and concentrated to 1–10 mg/ml.

### Histone methyl transferase assays

For *in vitro* HMTase assays, SMYD3 proteins (0.1–1 μg) +/− equivalent amt. of human HSP90α (Assay Designs, Ann Arbor, MI, USA, cat. no SPP-776D) were incubated with 1 μg of mixed histones from calf thymus (Sigma) or recombinant core histones (Upstate). Two μCi S-adenosyl-L–[methyl-^3^H] methionine (SAM; Amersham Biosciences) was included as a methyl donor. All reactions were carried out in 40 μl HMT reaction buffer (10 mM dithiothreitol, 100 mM NaCl, 4 mM MgCl2, and 50 mM Tris-HCl at pH 8.8) at 30°C for 3 hours. An 18% SDS-PAGE gel was used to resolve samples and fluorography was used to visualize isotope incorporation. Substrate loading was visualized by Coomassie blue staining.

### HSP90 and GST-MEEVD binding assays

Determination of apparent dissociation constants (K_d_) values for wildtype or mutant 6X-His-SMYD3 with either HSP90α or GST-MEEVD (plasmid provided by Dr. Lynne Regan, Yale Univ.) complex formation was carried out as follows: 1.5 μM of each purified 6X-His-SMYD3 protein was mixed with various amounts of HSP90α or GST-MEEVD (0.25, 0.5, 1, 2, 4, 8, 16, and 32 μM) in 130 μl of buffer B (20 mM Tris-HCl (pH 8.0), 300 mM NaCl, 20 mM imidazole, 5 mM β-mercaptoethanol, and 5% glycerol) in the presence or absence of 1 mM ATP plus 5 mM MgCl2 and incubated at 25°C for 30 min. Ni-NTA-agarose (15 μl of a 50% slurry in buffer B, Qiagen) was added to each reaction mixture, and incubation was carried out at 4°C with constant shaking for 40 min. Mixtures were transferred to an Ultrafree-MC centrifugal filter device (UFC30HV00, Millipore) and centrifuged at 6000 rpm for 10s. After 3 washes with Buffer B, the Ni-NTA-agarose was pelleted at 6000 rpm for 10 s. Resin was then mixed with 10 μl of elution buffer [20 mM Tris-HCl (pH 8.0), 300 mM NaCl, 5 mM β-mercaptoethanol, 250 mM imidazole, and 5% glycerol] and incubated at room temperature for 10 min. Following centrifugation at 6000 rpm for 1min, elution step was repeated and combined eluates were fractionated on SDS-PAGE. For input controls, 10% of the amounts of HSP90α and GST-MEEVD used for binding reactions were processed identically but in the absence of 6X-His-SMYD3 proteins. After staining with Coomassie Blue, protein amounts were quantitatively estimated with a densitometer (GS-800™, Bio-Rad). Ratio of densities of HSP90 or GST-MEEVD to 6X-His-SMYD3 represents the percentage of 6X-His-SMYD3 bound. Concentration of 6X-His-SMYD3 /HSP90 and 6X-His-SMYD3/GST-MEEVD complexes were derived from the ratio of their densities multiplied by total 6X-His-SMYD3 concentration (1.5 μM). Concentrations of respective complexes were plotted against total concentrations of HSP90 or GST-MEEVD. K_d_ values were obtained by non-linear least square curve fitting using the Sigmaplot program (SSPS Inc.) using the following equation:

ER = (K_d_ + Et + Rt) ─ √(K_d_ + Et + Rt)^2^ ─ 4 X Et + Rt)/2, where ER is the concentration of the 6X-His-SMYD3-HSP90 or GST-MEEVD complex; Et, total HSP90 or GST-MEEVD concentration; and Rt, total SMYD3 concentration.

### Mammalian cell transfection and western blotting

Wildtype and mutant SMYD3 cDNAs were transferred from Gateway pENTR into pEF-DEST51 (N-terminal V5-tagged) by TOPO cloning. NIH3T3 cells were transiently transfected, harvested 48 hours later, and then lysed in RIPA buffer (150 mM NaCl, 1% NP-40, 0.5% DOC, 50 mM Tris pH 8, 0.1% SDS) containing protease inhibitors (Roche Molecular Biochemicals, Indianapolis, IN). Expression levels were determined by Western blotting. Proteins were resolved on 8–15% SDS-PAGE, transferred to nitrocellulose (Protran BA, Schleicher and Schuell, NH), and blocked using 5% nonfat milk (10g nonfat milk, 150 mM NaCl, 10 mM Tris pH 8, 0.05% Tween-20) overnight at 4°C. Membranes were incubated with anti-SMYD3 polyclonal antibody [19] for 1 hour at room temperature, extensively washed, then incubated with ECL Plex Goat anti-Rabbit IgG-Cy5 Secondary Antibody (GE Healthcare) for 1 hour at room temperature. Blots were exposed and developed using ECL blot detection reagent (Amersham Pharmacia Biotech) according to manufacturer's instructions.

### Proliferation assays

Mouse embryonic fibroblasts (MEFs) were isolated from E13.5 C57Bl/6 embryos as previously described [49]. Cells were plated at ~5 × 10^6^/ml in RPMI (supplemented with 10% heat-inactivated fetal calf serum, 100 U/ml penicillin, 100 μg/ml streptomycin, 5 × 10^−5^ M β-mercaptoethanol, and 1 mM sodium pyruvate)transfected) and transfected by lipofection (Fugene) with ~6 ug of either SMYD3 wildtype, mutants or empty vector. To determine the rates of cell proliferation, transfected MEFs were plated in triplicate 1 d after infection at a density of 10^4^ cells/cm^2^ and counted every 24 h using a Z1 Coulter Particle Counter (Beckman Coulter) with elimination of dead cells calculated by trypan blue exclusion.

### Cell fractionation

Cells were separated into cytoplasmic (C), soluble nuclear protein (NP), chromatin (CH), and nuclear matrix (NM) fractions as follows. Approximately 1 × 10^8^ cells were washed twice in PBS and the pellet was resuspended in 2ml HNB buffer (500 mM sucrose/15 mM Tris-HCL pH 7.5/60 mM KCL/.25 mM EDTA/.125 mM EGTA/.5 mM spermidine). Then 1ml HNBN buffer was added dropwise (HBN buffer+ 1% NP-40) and incubated at 4°C for 5 minutes before centrifugation at 6,000g for 3 min at 4°C; the supernatant of this is the C fraction. The pellet was then resuspended in 1ml CSKT buffer (CSK buffer + 1% Triton-X), incubated at 4°C for 5 minutes before centrifugation at 3,000g for 3 min at 4°C; the supernatant of this is the NP fraction. The pellet was then resuspended in 720 μl CSK buffer (10 mM Pipes pH 6.8/300 mM sucrose/3 mM MgCl_2_/2 mM EGTA) and 30 μl RNase-free DNase, incubated at 37°C for 15 minutes then added 250 μl 1M AmSO_4_/CSK and incubate at 4°C for 5 minutes, before centrifugation at 3,000g for 3 min at 4°C; the supernatant of this is the CH fraction. The pellet was resuspended in 1ml 8M Urea and centrifuged at 13,000g for 5 minutes; the supernatant is the NM fraction. Purity of the subfractions was assessed by western blotting with antibodies noted in Figure 7C as previously described [50].
